# Hemodynamic Characteristics of the Vertebrobasilar System Analyzed Using MRI-Based Models

**DOI:** 10.1371/journal.pone.0051346

**Published:** 2012-12-10

**Authors:** Amanda K. Wake-Buck, J. Christopher Gatenby, John C. Gore

**Affiliations:** 1 Department of Radiology and Radiological Sciences, Vanderbilt University, Nashville, Tennessee, United States of America; 2 Department of Radiology, University of Washington, Seattle, Washington, United States of America; 3 Department of Biomedical Engineering, Vanderbilt University, Nashville, Tennessee, United States of America; 4 Department of Molecular Physiology and Biophysics, Vanderbilt University, Nashville, Tennessee, United States of America; 5 Department of Physics and Astronomy, Vanderbilt University, Nashville, Tennessee, United States of America; Sapienza University of Rome, Italy

## Abstract

The vertebrobasilar system (VBS) is unique in human anatomy in that two arteries merge into a single vessel, and it is especially important because it supplies the posterior circulation of the brain. Atherosclerosis develops in this region, and atherosclerotic plaques in the vertebrobasilar confluence can progress with catastrophic consequences, including artery occlusion. Quantitative assessments of the flow characteristics in the VBS could elucidate the factors that influence flow patterns in this confluence, and deviations from normal patterns might then be used to predict locations to monitor for potential pathological changes, to detect early signs of disease, and to evaluate treatment options and efficacy. In this study, high-field MRI was used in conjunction with computational fluid dynamics (CFD) modeling to investigate the hemodynamics of subject-specific confluence models (n = 5) and to identify different geometrical classes of vertebrobasilar systems (n = 12) of healthy adult subjects. The curvature of the vessels and their mutual orientation significantly affected flow parameters in the VBS. The basilar artery geometry strongly influenced both skewing of the velocity profiles and the wall shear stress distributions in the VBS. All five subjects modeled possessed varying degrees of vertebral asymmetry, and helical flow was observed in four cases, suggesting that factors other than vertebral asymmetry influence mixing of the vertebral artery flow contributions. These preliminary studies verify that quantitative, MR imaging techniques in conjunction with subject-specific CFD models of healthy adult subjects may be used to characterize VBS hemodynamics and to predict flow features that have been related to the initiation and development of atherosclerosis in large arteries. This work represents an important first step towards applying this approach to study disease initiation and progression in the VBS.

## Introduction

The vertebrobasilar system is unique in humans in that it is the sole instance where two large arteries (the vertebral arteries) merge into another vessel (the basilar artery). Located proximal to the Circle of Willis, the basilar artery is the primary supply to the posterior brain. Manifestations of disease in this confluence, including vertebrobasilar dissection and occlusion, can have significant consequences. Although it accounts for only approximately 1% of all ischemic strokes, acute basilar artery occlusion (ABAO) occurs in younger patients (mean age 60 vs. 71 years in all-cause ischemic stroke) and results in greater risk for in-hospital death than all other causes of ischemic stroke (30% vs. 8% in-hospital mortality) and generally poorer functional outcomes (in nearly 80% of surviving patients) upon release [1], [2].

The vertebrobasilar confluence is not geometrically as typical a location for developing atherosclerosis as are bifurcations or curves, but Ravensbergen *et al.* [3] identified plaque at the apex of the VBS in 50% of human cadavers examined. Of the 11 vertebrobasilar systems with plaque that they investigated in-depth, nine cases were severe. The integrated plaque thickness along the basilar artery at the lateral walls was significantly greater than that at the anterior and posterior walls of the vessel, and the plaque thickness decreased along the basilar artery. When one vertebral artery was larger than the other, the plaque thickness on the lateral wall of the basilar artery was greater on the same side as the dominant vertebral. This statistically significant difference in the circumferential localization of basilar artery plaque implicates a role for hemodynamics in the development of atherosclerosis at this location. Moreover, in other vasculature, local flow characteristics such as areas of low and oscillating wall shear stress (WSS) and recirculation regions correlate strongly with atherosclerosis initiation, development, and vulnerability [4]–[7].

Previous hemodynamic studies investigated flow in idealized, planar, vertebrobasilar systems, analyzing the effect of asymmetry (diameter or flow rate differences) between the contributing vertebral vessels and of confluence shape and angle on the flow field [8]–[11]. Vessels excised from elderly cadavers (mean age = 81) were used by Kobayashi and Karino [12] in *in vitro* flow models that characterized streamlines and velocity profiles in the VBS in response to different steady flow rates. In addition, an MR angiography study observed a relationship between vertebral size and flow mixture in the basilar artery [13]. Although these differences explain some large-scale flow patterns in vertebrobasilar systems with relatively simple, planar geometries, they are insufficient for describing the time-resolved flow fields in real vessels with three-dimensional geometries, and there remains a need for a non-invasive quantitative characterization of hemodynamics in healthy, younger adult subjects, particularly with respect to pathophysiologically-relevant parameters.

Clinical imaging-based computational fluid dynamics (CFD) simulations previously have been used to successfully evaluate hemodynamics and can provide three-dimensional descriptions of flow fields. (For a thorough review, see Steinman [14].) In addition, imaging-based CFD simulations can be incorporated into surgical planning [15], [16]. Multi-directional MR velocimetry and CFD have been used together to characterize hemodynamics in patients with basilar aneurysms and in basilar tip aneurysm models, and they demonstrate the feasibility of using CFD with MR imaging to study this system [17]–[21].

In this study, we used quantitative high-field MR imaging techniques in conjunction with subject-specific CFD models of healthy adult subjects to characterize hemodynamics in the VBS and to identify flow features that are related to atherosclerosis initiation and development in bifurcations. In addition, we compared geometric variations across subjects and used these to refine descriptions of different vertebrobasilar configurations. Previous patient-specific models have investigated flow in basilar aneurysm models, but the relationship between atherosclerosis development and hemodynamics in this arterial confluence remains to be fully elucidated. Understanding the precise characteristics of flow in the healthy VBS will provide the knowledge base needed for future studies designed to elucidate the factors that affect flow patterns in this system. One major long-term aim is to identify and distinguish variations in these patterns in order to predict potential locations to monitor for pathology, identify early signs of pathology, determine prognosis, and evaluate treatment options or efficacy.

## Materials and Methods

**Figure 1:**
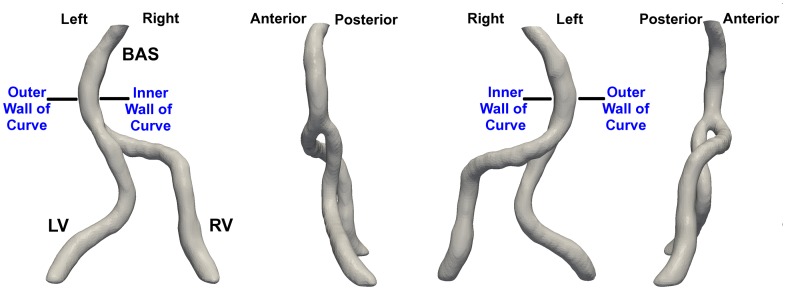
Vertebrobasilar configuration. Representative vertebrobasilar configuration shown at 90-degree rotations. The direction of the flow is from the right vertebral (RV) and left vertebral (LV) arteries into the basilar artery (BAS). Anatomical orientation is labeled at the top. Since the BAS curvature direction is not preserved between subjects and the curve direction influences the flow field, the results and analysis refer to the “inner” and “outer” walls of the curve *in lieu* of standard anatomical directions.

This study was approved by the Vanderbilt University Institutional Review Board, and signed informed consent was obtained from all subjects prior to their participation in the study. A Philips Achieva 3T MR system (Philips Healthcare) and 16-channel SENSE neuro-vascular coil were used to acquire geometric data in 14 healthy, adult subjects. Two of these subjects did not have a right vertebral artery joining the confluence (i.e. the right vertebral artery terminated in a posterior inferior cerebellar artery (PICA)) and were excluded from this analysis. In five subjects (A–E), subject-specific computational CFD models were built (Figure 1), and the geometry and pulsatile flow boundary conditions were determined from time-of-flight (TOF) [22], [23] and phase contrast MR (PCMR) [24], [25] axial images, respectively, as described below.

### MR Imaging

#### MR-based geometry acquisition

The geometry of the vertebrobasilar confluence was evaluated using three-dimensional TOF angiography. The TOF data also were used to define measurement planes for subsequent PCMR velocity measurements. Scan parameters were: field of view (FOV) = 160 mm×80 mm; number of slices = 100 (for B, 110 slices were used to cover the confluence geometry); acquired voxel size = 0.5 mm×0.5×0.6 mm; reconstructed voxel size = 0.4 mm×0.4 mm×0.6 mm (for B, reconstructed voxel size was 0.4 mm×0.4 mm×0.7 mm); repetition time (TR) = 23 ms; (echo time) TE = 3.45 ms; flip angle = 20°; sensitivity encoding (SENSE) factor = 2. For each subject, 36 maximum intensity projections (MIPs) were created at 5° axial rotation increments using the Philips console graphical user interface to facilitate three-dimensional assessment of the geometries and to orient the subsequent PCMR volumes.

#### MR flow quantification

To measure flow in the left and right vertebral arteries in five of the subjects, PCMR acquisition planes were oriented perpendicular to the vessel axes. Retrospectively electrocardiogram- (ECG-) or peripheral pulse unit- (PPU-) triggered PCMR imaging was used to measure the time-varying, through-plane velocity distribution across the vessel lumen at 21 or 25 time points per cardiac cycle in each of the vertebral arteries. Scan parameters were: FOV = 100 mm×100 mm; acquired voxel size = 0.33 mm×0.33 mm×5 mm; reconstructed voxel size = 0.31 mm×0.31 mm×5 mm; TR = 15 ms; TE = shortest, ∼8.5 (21 phases) or 8.2 (25 phases) ms; velocity encoding value (VENC) = 100 cm/s.

### Computational Fluid Dynamics Modeling

#### Geometry boundary conditions

Using Amira 5.2.0 (Visage Imaging, Inc.), small, spurious branches were masked from the TOF data set. The vessel lumen of the vertebrobasilar confluence was segmented from the masked TOF data and reconstructed using ITK-SNAP 2.0.0 (itksnap.org) [26]. The reconstructed vessel surface was smoothed using a Taubin filter in Meshlab v.1.2.1 (Visual Computing Lab, Instituto di Scienza e Tecnologie dell’Informazione, Consiglio Nazionale delle Ricerche, Italy; http://meshlab.sourceforge.net/). The resulting surface was imported into GAMBIT (Ansys, Inc.), in which a computational volume was constructed by capping the ends of the vessels. Entrance and exit lengths were extruded from the vertebral and basilar vessels at distances that allowed the flow to be fully developed upon entrance into the vessel system at peak flow rates. The volume was discretized using tetrahedral elements.

#### Flow boundary conditions

**Figure 2:**
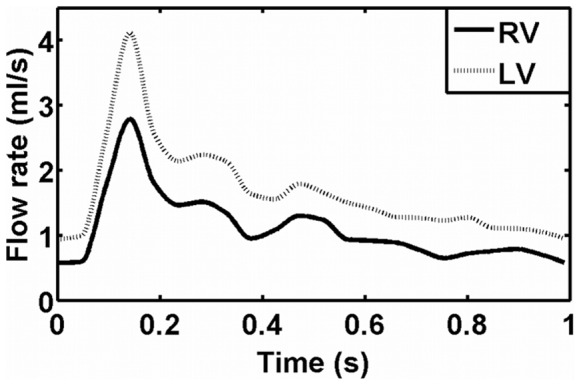
Vertebral artery flow waveforms. Representative mass flow (ml/s) waveform (determined for C). Solid line: right vertebral (RV) flow; dotted line: left vertebral (LV) flow.

The PCMR data from the left and right vertebral arteries were segmented using Amira 5.2.0. Using MATLAB v.7.8.0 (Mathworks, Inc.) the through-plane velocity values were interpolated across the segmented vessel lumen to calculate the flow rate at each time point. Piecewise continuous interpolation of the vessel flow rates between the PCMR-measured time points produced a pulse waveform described at 500 points over the cardiac cycle (Figure 2). The resulting waveforms were assigned as unsteady mass flow inlet boundary conditions in the flow simulations.

#### Simulations

All simulations were conducted using the commercially available CFD code Fluent 12.0.16 (Ansys, Inc.). The rigid wall assumption was used; the no-slip boundary condition was imposed at the vessel wall; and the basilar artery outlet was prescribed traction-free. The fluid was assumed Newtonian, and fluid parameters were chosen to be consistent with values for blood (ρ = 1053 kg/m^3^, μ = 0.00368 kg/m-s). The Newtonian fluid assumption is acceptable for flow in large arteries, and the Newtonian assumption was shown to impart the lowest order of impact on simulation results [4], [27], [28]. For each vessel system, steady simulations were conducted at the peak systolic incoming flow rates for that system (i.e. the inlet mass flow boundary conditions of the LV and RV were the PCMR-measured values at peak systole) to determine grid independence of the model. Increasing the number of tetrahedral grid elements in these models by roughly 50% resulted in mean differences in peak axial velocity and axial WSS of 2.3% and 3.9%, respectively, confirming grid independence.

For each model, steady state simulations were conducted with boundary conditions determined from the initial PCMR data time point (t = 0), and the simulations were run to convergence. Pulsatile simulations for the pulse cycle were initiated from the steady state results, and each simulation contained 500 time steps over the course of the pulse cycle. The pulsatile simulations were conducted over three pulse cycles (1500 time steps) to verify convergence.

#### Analysis

The wall shear stress data were averaged over the final 500 time steps (the last pulse cycle simulated) using MATLAB. The oscillatory shear index (OSI) was calculated using the definition of He and Ku (1996) [29]. Time-averaged WSS and OSI results were visualized using Tecplot 360 2011 (Tecplot, Inc.), and all other results were visualized using Fluent. Due to the large data set, we present data from time points in each subject-specific simulation that correspond only to key features in the cardiac cycle: peak systolic acceleration, mid-systolic deceleration, and end diastole. Given the variation of basilar artery curve direction among the subjects and the influence of this basilar artery curvature on the local flow field, we refer to the “inner” and “outer” walls of the basilar curve for orientation *in lieu* of standard anatomical directions for clarification (Figure 1).

## Results

### Geometric Variations

**Figure 3:**
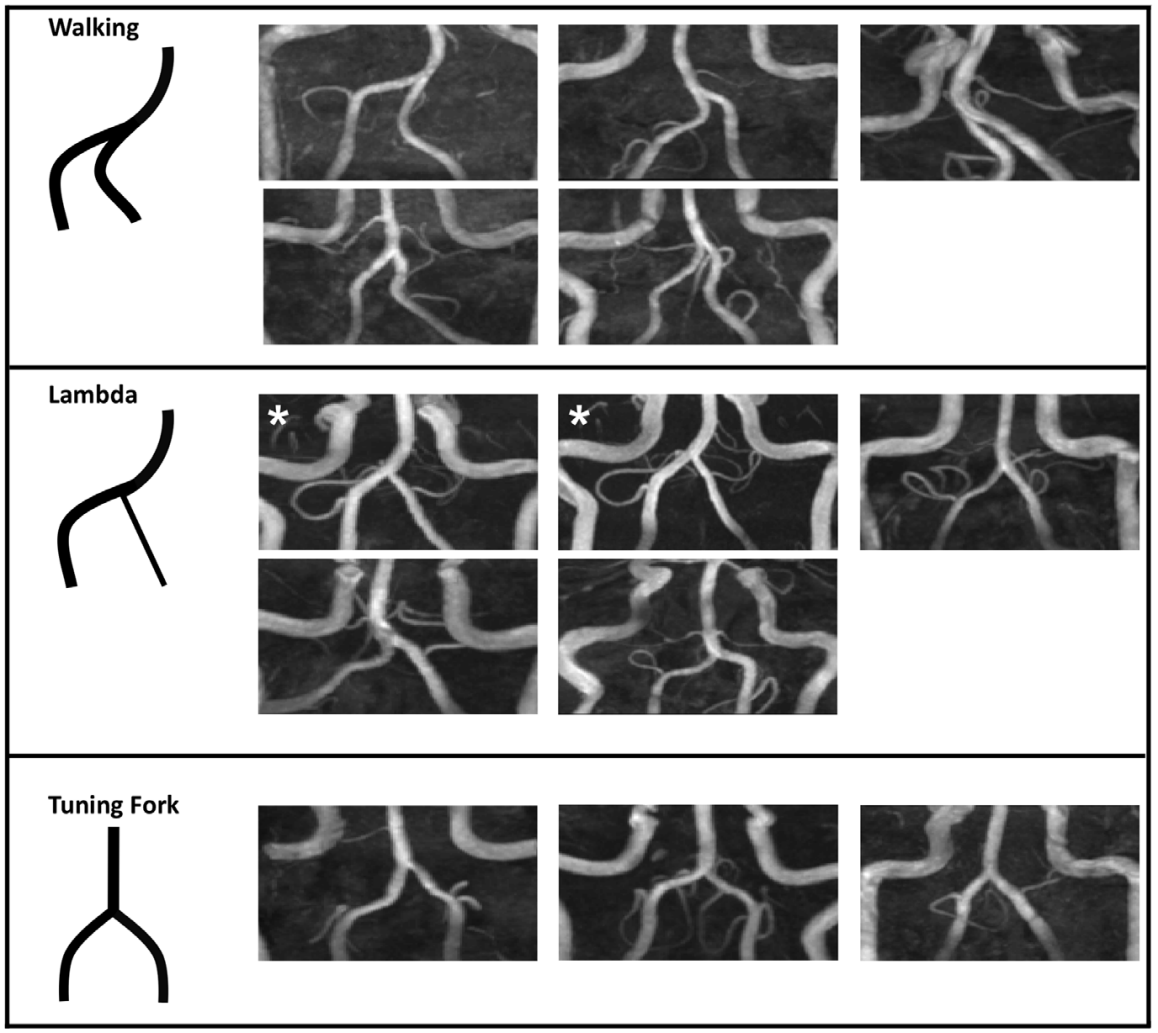
Vertebrobasilar confluence geometries. Vertebrobasilar system geometries of 12 healthy young adult subjects are divided into three classifications: Walking, Lambda, and Tuning Fork. In each panel, a schematic of the configuration is followed by anterior-posterior MIPs of the subject data. The two images marked with asterisks show the same subject imaged on two different days, demonstrating that the vessels retain their geometric configuration with repositioning.

MR images of 12 healthy young adults demonstrated variations between subjects. Based on the TOF images, we found VBS geometries can be qualitatively classified into three basic geometric configurations: Walking (five subjects, including A and B), Tuning Fork (three subjects, including C and D), and Lambda (four subjects, including E) (Figure 3). The Walking geometry is distinguished by vertebral arteries that bend in the same direction (e.g. left or right) before merging into the basilar artery. The Tuning Fork configuration exhibits vertebral arteries that join at the confluence at rather symmetrical angles with respect to the basilar artery; the feeding arteries can be bent in opposite directions. In the Lambda configuration, the basilar artery path follows the direction dictated by one of the vertebral arteries (primary or dominant vertebral); the other vertebral artery, typically smaller, abuts the primary vertebral/basilar artery path, forming a pseudo T-junction. Interestingly, Hong *et al.* [30] reported vertebral dominance in conjunction with a basilar curve in the opposite direction in 83.5% of patients, which appears to describe the Lamba geometry. The two asterisks in Figure 3 identify the same subject imaged on different days, demonstrating reproducibility of the geometric configuration.

### Flow Rates

**Figure 4:**
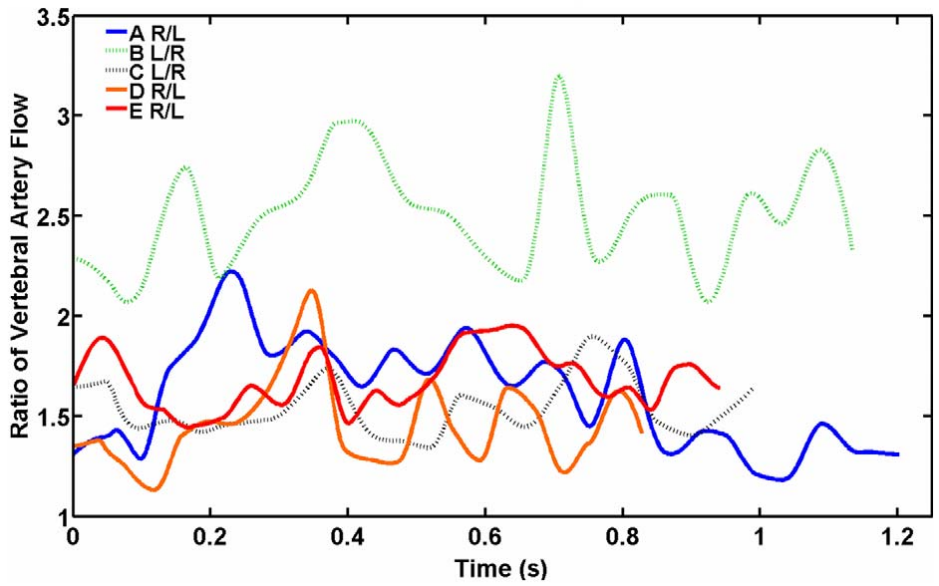
Vertebral artery flow ratios. The time-varying vertebral artery flow ratio over the pulse cycle for the five healthy young adult subjects. Solid lines represent the ratio of right vertebral (RV) to left vertebral (LV) flow (i.e. RV/LV); dotted lines represent the ratio of LV to RV flow (i.e. LV/RV).

Typical PCMR-measured RV and LV flow rates used as inflow boundary conditions are presented in Figure 2. Figure 4 shows the ratio of incoming flows over the pulse cycle for each subject, which vary considerably both inter- and intra-subject. The ratio of incoming flows ranged from approximately 1.1 (RV:LV for D) to 3.2 (LV:RV for B) (Table 1), and two subjects (C, E) exhibited flow ratios that were rather consistent over the pulse cycle (i.e. span of 0.51 for E, 0.56 for C). In A, E, and D, the RV flow is greater than that of the LV throughout the pulse cycle by approximately 1.1- to 2.2-fold. At peak systole, the ratio of incoming flows is 2.2 for A (Table 1); however, for D and E, the ratios of incoming flows at peak systole (1.45 and 1.13, respectively) are much less than the maximum ratios, which occur after peak systole. In B and C, the LV flow is greater than that of the RV throughout the entire pulse cycle with an LV:RV ratio ranging from 1.3 to 3.2, with ratios at peak systole of 2.2 and 1.47, respectively.

**Table 1:** Pulse cycle characteristics.

	A	B	C	D	E
**Vertebral flow ratio minimum and** **maximum**	1.18–2.22	2.07–3.20	1.34–1.90	1.13–2.13	1.44–1.95
**Vertebral flow ratio at peak systole**	2.22 RV:LV	2.22 LV:RV	1.47 LV:RV	1.13 RV:LV	1.13 RV:LV

Characteristics of the pulse cycle of each subject as determined from phase contrast MR (PCMR); RV = right vertebral artery; LV = left vertebral artery.

### Velocity Profiles

**Figure 5:**
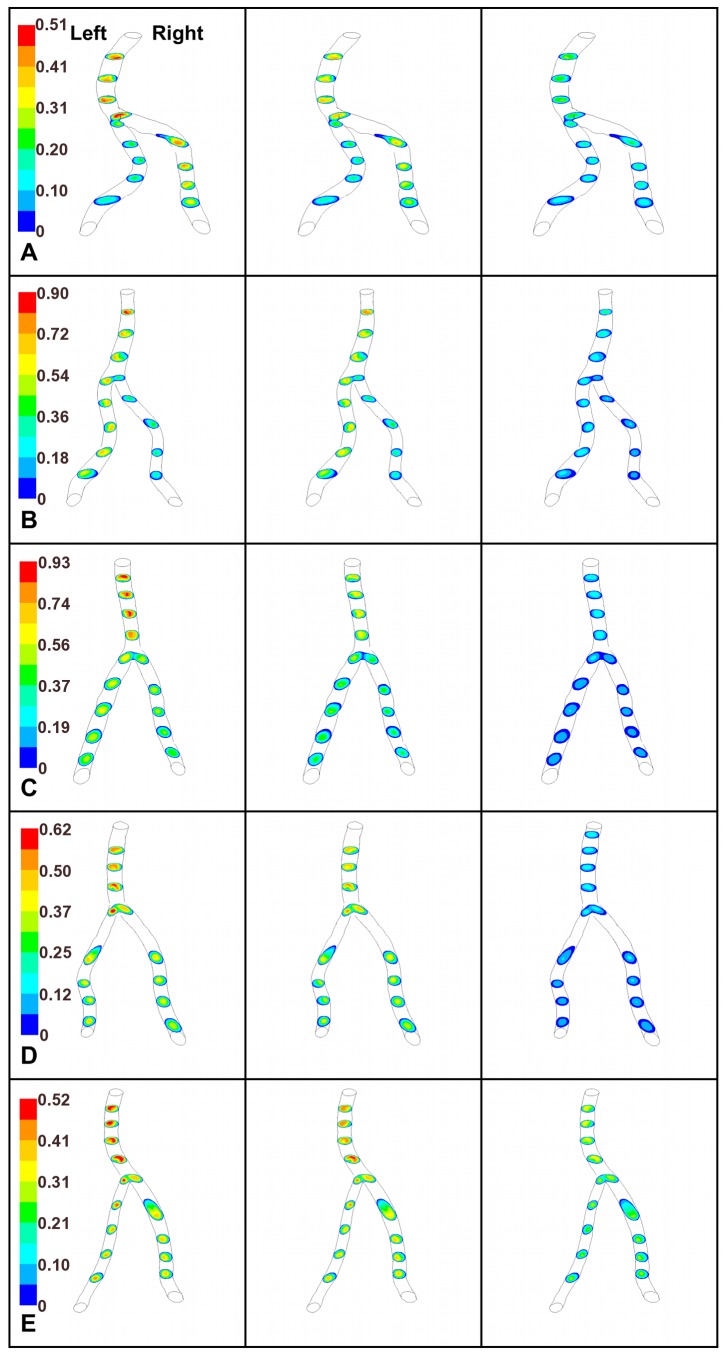
Axial velocity profiles. Axial velocity profiles at several axial planes along the vertebrobasilar confluence of five subject-specific models. For each model the time points shown are peak systole (left column), systolic deceleration (middle column), and diastole (right column). Colorbar representing velocity (m/s) is given for each subject.

Velocity contours from the CFD calculations are presented at different slice locations along the VBS (Figure 5). In all subjects, the velocity profile patterns shown for the peak systole time point are qualitatively similar to the patterns in systolic deceleration; however, the profiles during systolic deceleration tend to be slightly flatter. In all models, the proximal basilar region contains two velocity peaks, which originate from the two vertebral arteries; these dual peak velocity regions quickly merge into a single peak along the basilar artery. The velocity profiles during diastole are flat compared to those during systole, so our report focuses on the velocity contours at peak systole.

**Figure 6:**
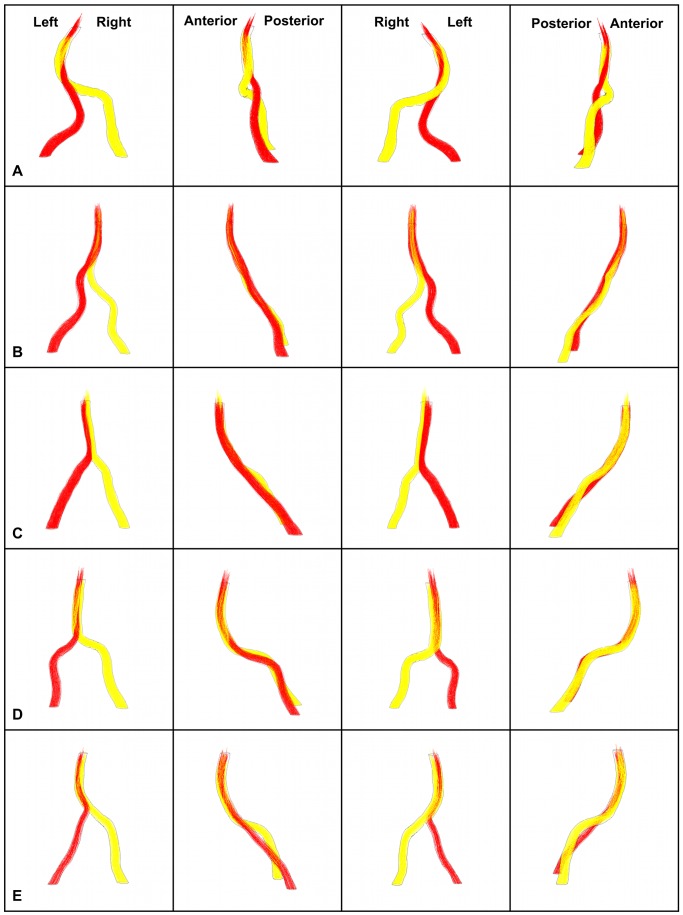
Pathlines. Pathlines colored by vertebral artery of origin at the CFD time point closest to peak systole for the five adult subjects at four 90-degree rotations. The orientations in the top row are applicable to all rows. Pathlines originating from the RV are yellow; those from the LV are red.

Vessel curvature influences velocity distributions in the basilar artery of these models. In each of the confluences modeled, the velocity peak predominantly skews toward the outer wall of the basilar artery curve, particularly during systole. This concentration of high momentum fluid fluctuates over the pulse cycle and shifts along the length of the basilar artery.

**Figure 7:**
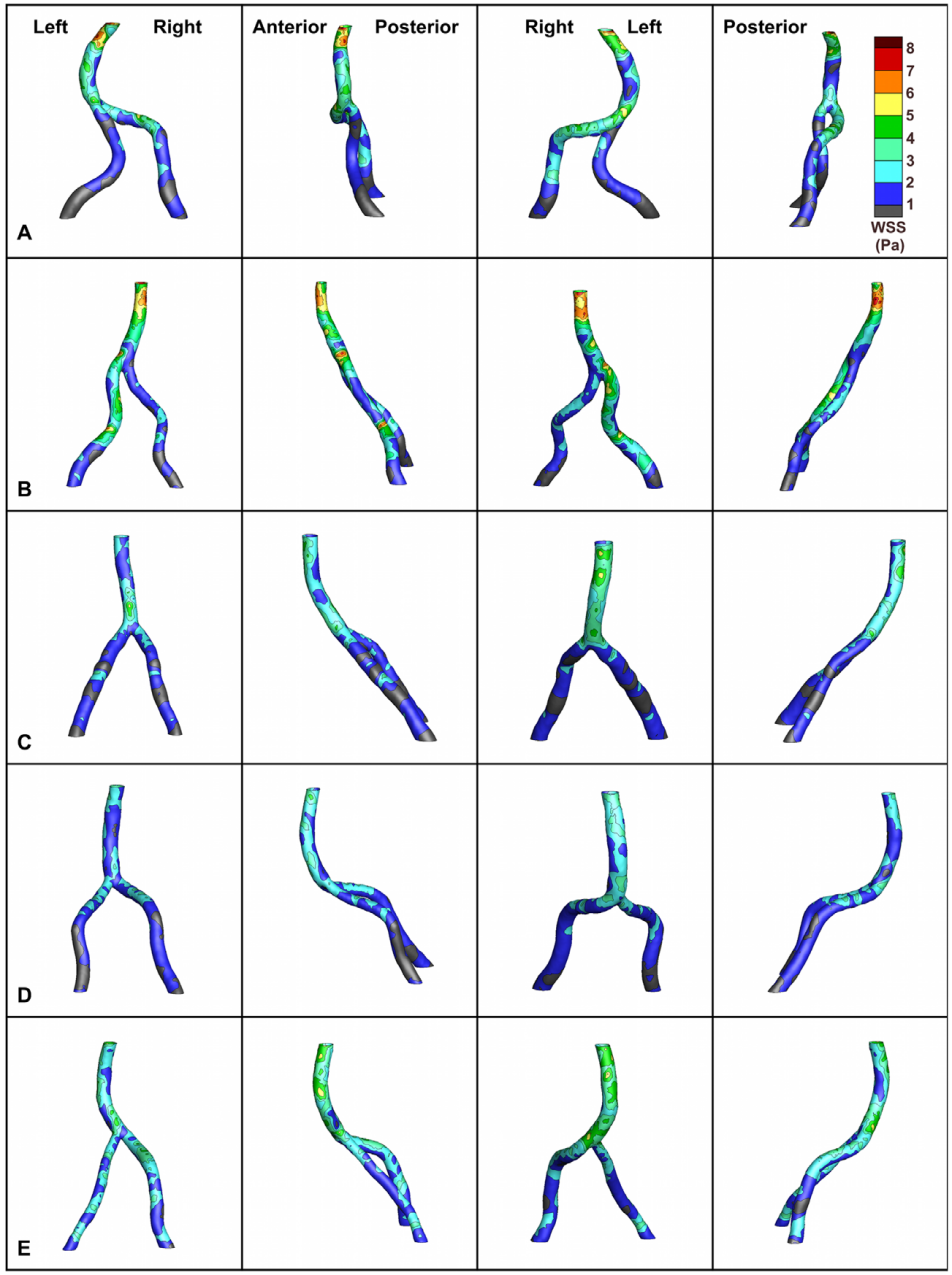
Time-averaged wall shear stress distributions. Time-averaged wall shear stress (WSS) for each vertebrobasilar system at four 90-degree rotations. The orientations and colorbar (WSS in Pa) in the top row are applicable to all rows.

**Figure 8:**
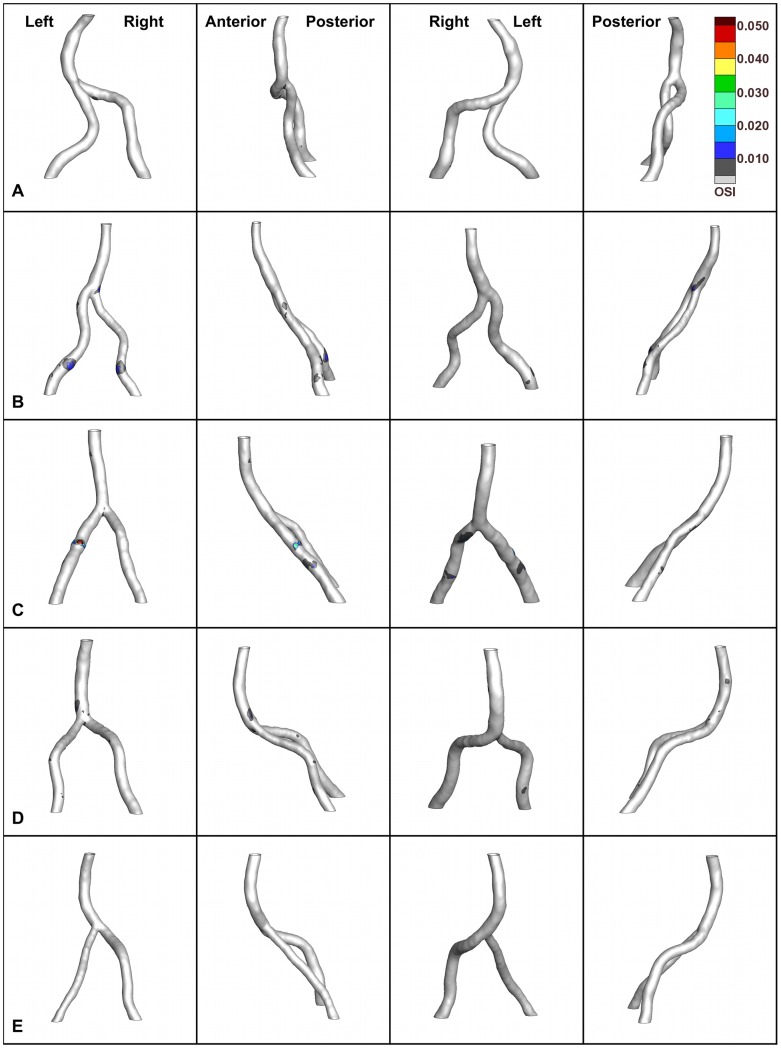
Oscillatory shear index contours. Oscillatory shear index (OSI) contours for each vertebrobasilar system at four 90-degree rotations. The orientations and colorbar in the top row are applicable to all rows. For E, OSI ≤0.005; however, the model is included for completeness.

### Walking Geometry

In both of the Walking geometries (A and B) the basilar artery direction continues out of the arcs formed by the vertebral arteries (i.e. the vertebral arteries converge while both sweep to the left); therefore, vertebral artery curvature proximal of the confluence influences velocity profiles in the basilar artery. Consequently, the high velocity region in these Walking geometries resides along the left side of the confluence. This effect is most evident in A, which also exhibits strong basilar artery curvature. Additionally, the ratio of flow from the contributing arteries in conjunction with the direction of vertebral artery approach influence velocity profile shape. As seen in A, flow from the RV, which is greater than that of the LV, impinges on the left wall of the basilar artery, swirling upward and causing the velocity profile to skew to the outer surface of the basilar artery curve (Figure 5). This RV flow direction forces the core LV flow in the proximal basilar to the posterior wall, resulting in slight posterior displacement of the maximum velocity peak. Unlike the other Walking geometry, the LV flow in B is greater than the RV flow and the basilar artery is relatively straight. Therefore, the double-peaked velocity profile in the confluence merges distally into a single peak that resides along the left wall of the basilar artery due to the continuation of the LV core flow, and this single peak spreads along the posterior wall of the distal basilar, suggesting that inflow ratio and vessel curvature collectively influence flow patterns.

#### Tuning fork geometry

The basilar artery curves in both the Tuning Fork geometries (C and D). This curvature causes the maximum velocity region to localize at the outer wall of the basilar artery curve. Velocity profiles in both the Tuning Fork geometries (C and D) are influenced by the basilar curvature. In C, the basilar artery is rather straight until the most distal portion, at which point it curves; consequently, the flow is roughly parallel and the velocity profile peak is rather central in the proximal basilar artery but evident along the anterior side at the distal portion. In contrast, the basilar artery of D is strongly curved, and the high momentum fluid resides along the curve’s outer wall.

#### Lambda geometry

In the Lambda geometry (E), velocity distributions are predominantly influenced by basilar artery curvature. In E, although the RV is the larger of the contributing arteries, the peak velocity skews toward the right-anterior wall of the proximal basilar artery because the high momentum core of the LV impacts the RV flow, shifting the velocity peak to the right-anterior side in the confluence and proximal basilar artery. More distally, the basilar artery curvature heavily influences the form of the axial velocity profiles in the Lambda geometry (E), and the high momentum fluid resides near the outer wall of the basilar artery curve. These results further support our findings that inflow ratio and vessel curvature synergistically affect local flow patterns.

### Pathlines

Pathlines, colored by the vertebral artery of origin, were determined for the five simulations (Figure 6). The pathlines shown are for the simulation time point just prior to peak systole; however, the patterns were qualitatively similar throughout the cardiac cycle. The vertebrobasilar systems in which the RV flow rate is larger than that of the LV (A, D, and E) exhibit flow from the RV (yellow) swirling around flow originating from the LV (red), consequently pushing the LV flow to the inner wall of the proximal basilar curve. In E this inner basilar curve LV flow is slightly shifted to the left side of the basilar. As the vertebral arteries join in B, in which LV flow is greater than RV by 2.2-fold, flow from the LV (red) pushes RV flow (yellow) toward the right side, encompassing the RV flow distally. In C (LV flow>RV flow), the flows from the contributing vertebral arteries do not mix in the basilar artery but remain on the same side as their artery of origin.

### Average WSS and OSI

Local regions of low WSS (defined as WSS<1 Pa) occurred at the crux of the junction in all subjects, and these low WSS foci develop even when there exists locally high WSS banding around both feeding arteries just proximal of the junction (e.g. A). In this region, high momentum streams from the vertebral arteries combine in the center of the basilar lumen, thus forcing high momentum fluid away from the junction of the feeding arteries. Further, the sudden expansion of the cross-sectional area causes a rapid decrease in the WSS at the junction apex. Additional low WSS regions reside on the inner wall of the basilar artery curve (A, C, D, and E), on the inner wall of the curve formed at the junction of the non-dominant vertebral artery and the basilar artery (B and E), and along the vertebral arteries.

Areas of locally high, time-averaged WSS occur at the outer wall of the primary curve of the basilar artery in four of the five models (Figure 7). Additionally, in the same four models (A, C, D, and E) there exists a region of locally high WSS on the inner wall of the basilar artery curve. The exception was B, the basilar artery of which was relatively straight; however, high WSS regions did develop on the left side of the LV (the dominant vessel) and on the outside of the curve formed by the vertebral arteries as they merge.

Overall, the confluences classified as Walking geometries exhibited higher time-averaged WSS values than the other geometries (A: 9.2; B: 8.3 Pa). The Lambda geometry had the median value (E: 6.2 Pa), and the lowest maximum time-averaged WSS values were predicted in the Tuning Fork models (C: 5.6; D: 4.5 Pa).

The highest OSI was seen in C (0.359), followed by D (0.023), B (0.015), A (0.009), and E (0.004) (Figure 8). A small fraction of the vessel surfaces demonstrated OSI greater than 0.005 (none of the surface for E). However, foci of OSI ≥0.005 coincided with lower WSS regions, particularly at or near the confluence apex (except E).

## Discussion

As demonstrated in Figure 3, even in the normal population considered in this study, the geometry of the vertebrobasilar confluence varies considerably but may be loosely classified into three types. In addition to varying LV and RV diameter asymmetries, the shapes of the apex and confluence vary widely between subjects. Previous reports determined that the LV was dominant (i.e. it had the larger flow rate or diameter) in 59–67% of subjects (mean age 81 and 62.5 years, respectively) [3], [30]. Further, Ravensbergen *et al.* [10] determined that the confluence angle of the VBS varies from 10–160° (Mean = 63°; SD = 30°) and reported that geometries were typically symmetric. In the VBS models presented here, the confluence angle range was 54–86° (Mean = 74°; SD = 14°), demonstrating that confluence angles in these subjects are within previously reported values even though the VBS angles could be classified as nearly symmetric in only two subjects. In spite of these variations, the 12 vertebrobasilar confluences observed in our study can be described by three geometric configurations: Walking, Lambda, or Tuning Fork.

Measurements of flow in the vertebral arteries demonstrate that the contributing flow ratios vary both throughout the pulse cycle and between subjects (Figure 4). Several previous studies investigated the impact of flow ratio on models of flow in non-diseased vertebrobasilar systems, and these experiments were conducted at steady flow or with idealized waveforms [8], [9], [12]. For models of healthy subjects presented in this study, we used MR-measured, subject-specific flow rates and evaluated the subject-specific flow ratios. The observed inter- and intra-subject variability in vertebral flow ratio is consistent with the findings of Ford *et al.* [31], who reported that vertebral flow rates vary substantially both inter and intra-subject, and with patient-specific velocity data presented by Rayz *et al*. [18]. It follows that flow rate variability would result in the flow ratio fluctuations observed herein.

### Velocity Profiles

Previous reports emphasize the effect of vertebral asymmetry on basilar artery velocity patterns. Just distal of the confluence, a double-peaked axial velocity profile was reported by Krijger *et al.* [9] in models of a planar, merging pair of channels with rectangular cross-sections and by subsequent studies. They demonstrated that for steady flow in both symmetric (equal flow in the vertebral arteries) and asymmetric (one vertebral artery has higher flow than the other) confluences an M-shaped axial velocity profile merges into one peak, which in the symmetric confluence develops and remains in the center of the basilar artery. In their asymmetric confluence, the M-shaped profile at the confluence is higher on the side that receives flow from the vertebral artery with greater flow, but along the length of the basilar artery the two peaks merge, and the maximum crosses the artery to skew toward the opposite wall before drifting back toward the center of the vessel. Previous groups investigating hemodynamics in *in vitro* vertebrobasilar models report that asymmetrical vessels or flow rate differences between contributing vessels induce helical flow in the basilar artery [12], [20]. Further, simulating flow in patient-specific basilar aneurysm models, Rayz *et al.* [18] reported that varying the vertebral artery inflow ratio profoundly affected flow patterns in the aneurysm.

In this study of subject-specific models, the influence of flow ratio appears to dominate in the most proximal portion of the confluence with contributions from basilar curvature influencing flow distal of the merge. Our CFD results suggest factors other than flow ratio are involved, including the curvature of the vessels and their orientation to each other, which can be addressed by analysis of their shape, particularly basilar artery curvature. Flow in a curved vessel generates a velocity profile peak near the outer wall of the curve due to a secondary flow in the cross-sectional plane (induced by an in-plane pressure gradient that balances the centrifugal force created by flow through the curve), as first investigated theoretically by Dean [32], [33]. This phenomenon appears to strongly influence the location of the peak velocity in our vertebrobasilar models.

#### Walking geometry

In both the Walking geometries (A and B), the basilar artery direction continues out of the arcs formed by the vertebral arteries; therefore, the vertebral artery curvature proximal of the confluence influences velocity profiles in the basilar artery. This effect is most evident in A, which also exhibits strong basilar artery curvature. In B, the LV flow is greater than twice the RV flow for the entire pulse cycle, yet the velocity profile in the basilar artery skews toward the left wall and remains on that side, contrasting with the contralateral peak or helical flow-induced shift reported previously [9], [12], [18].

#### Tuning fork geometry

A primary feature of the Tuning Fork geometry (C and D) is that the angles of approach between each vertebral and the basilar artery are roughly equal. The near symmetry of this geometric classification makes it most suitable for comparison with previous studies that used idealized or relatively symmetrical models. Previously, Chong *et al.* [20] attributed mixing in the confluence of *in vitro* flow models to contributing artery diameter differences, and Kobayashi and Karino [12] observed that comparable vertebral flow rates in a symmetrical geometry produced basilar flow with predominantly parallel flow and that degree of basilar artery flow mixing increased with the ratio of flow between the two vertebral arteries. Although D provides more symmetrical inflow conditions, the pathlines indicate flow mixing in the basilar artery and the velocity peak resides along the anterior wall, whereas in C the flow remains parallel for the length of the basilar artery. In the current investigation, the basilar artery curvature (which is strongly curved in D and which is rather straight until the distal portion of C) likely influences the basilar flow mixing and the shape of the axial velocity profiles in the Tuning Fork geometries.

#### Lambda geometry

The Lambda geometry characteristically consists of one vertebral abutting the other in a near T-junction, and typically there is a diameter difference between the vertebral arteries in this geometry. In E, the ratio of RV:LV flow is between 1.5 and 2.0 for the vast majority of the pulse cycle, and it exhibits behavior similar to that predicted in literature, where the maximum velocity peak would shift toward the left side of the basilar artery after merging of the two flow streams; this leftward shift is augmented by basilar artery curvature distal of the junction [9].

### Pathlines

In previous studies, large secondary flows were predicted in the region near the junction of idealized models with blunted apices, and this effect increased as the confluence angle decreased, corresponding to an increased likelihood of atherosclerosis in geometries with blunted apices at narrow angles [3], [11]. A manifestation of secondary flow is easily observed by spiral flow illustrated *via* vessel selective MR angiography or computed pathlines.

Of the basilar arteries they studied using MRA with selective saturation of the vertebral arteries, Smith and Bellon [13] characterized the flow in 80% (12 of 15) as parallel, and in 20% of the basilar arteries there appeared a spiral flow pattern. However, in vertebrobasilar systems with a dominant vertebral artery, the majority of the flow in both posterior communicating arteries was from the dominant artery, which suggests there is mixing of flow in the basilar artery, at least before its bifurcation. As described above, it was previously shown that asymmetry of flow influenced both the existence of flow crossing the sagittal plane of symmetry and the degree of helical flow, which decreases approximately two to six diameters distally [12], [18], [20]. Collectively, these results demonstrate an association between secondary flow along the basilar artery and unequal vertebral flow rates.

In this study, pathlines illustrate that the pattern of flow is more complicated than the helical flow described in the previous idealized or near planar models of flow and that, as described above, geometric factors (e.g. basilar artery curve, relative angle of approach of vertebral arteries) influence the secondary flow in the confluence. However, it is important to consider that the ratio between the vertebral inlet flows fluctuated throughout the cardiac cycle, consequently influencing quantitatively – if not qualitatively – the helical flow.

### Average WSS and OSI

Although pathlines and velocity profiles are useful for characterizing the flow field, it is important to investigate pathophysiologically-relevant flow parameters. The present study is the first to report WSS distributions in subject-specific models of the VBS of healthy young adult subjects. Previously, WSS distributions have been reported in idealized geometries and in patient-specific models of cerebrovascular pathologies [3], [17]–[21]. Our simulations predicted local low WSS regions at the apex of all models, corresponding to the Ravensbergen *et al.* [3] findings of plaques at the junction apex in 50% of cadavers. Ravensbergen *et al.* [10] reported that vertebrobasilar systems with atherosclerosis at the apex have a larger confluence angle. However, it is important to note that this observation implies correlation between confluence angle and atherosclerosis but not causation–indeed, atherosclerosis development may blunt the angle of the confluence.

Previously, steady flow in a planar confluence model demonstrated that WSS is lower along the lateral wall of the basilar artery on the same side as the dominant vertebral artery and that this low WSS region stretches the length of the basilar artery [3]. Further, they noted that in systems with vertebral asymmetry, the plaque thicknesses were greater on the side of the basilar artery that corresponded to the dominant vertebral artery. In contrast, our subject-specific simulations show local low WSS zones along the inner wall and high WSS foci along the outer wall mid-curve in four of the five basilar arteries (A, C, D, and E), suggesting a dependence on Dean’s-type flow. Due to centrifugal forces, flow in a curved pipe can cause the location of maximum axial velocity to shift toward the outer wall of a curve; this shift will produce areas of locally low WSS at the inner wall of the curve and high WSS at the outer wall of the curve, as demonstrated in the major curve of the basilar artery [32], [33]. In addition, these four models exhibit an area of locally high time-averaged WSS on the inner curve of the proximal portion of the basilar artery. This finding agrees with descriptions of entrance flow in a curved pipe, in which curvature previously was shown to induce velocity profile skewing and, consequently, locally high WSS values along the inner wall of proximal sections of a curve [34], [35]. The exception was B; however, Dean’s-type high WSS foci developed on the outside of the curve formed by the merging and curving vertebral arteries.

Additional low WSS regions formed at the junction of the non-dominant vertebral artery with the basilar artery in B and E. In the immediate vicinity of the confluence apex of these two models, the non-dominant artery angle of approach to the dominant vertebral and the basilar artery is reminiscent of the distal end-to-side anastomosis configurations used in previous studies [36]–[38]. The low WSS regions in the current models correspond to the area immediately distal of the “toe” region of the grafts, and it is widely recognized that in anastomoses this region experiences low WSS and/or flow separation and that intimal hyperplasia develops in this area [36]–[38]. This suggests that these focal WSS regions in the VBS might provide early indication of potential locations of atherosclerosis development.

Previous studies calculated mean WSS in other arterial locations from ultrasound-measured velocity profiles in subjects aged similarly to those in the current study [39]–[41]. They reported values of 0.30±0.16 to 0.42±0.27 Pa (several age groups were studied) in the common femoral artery [40], 0.5±0.2 Pa in the brachial artery [41], and 1.2±0.2 (females) to 1.3±0.3 Pa (males) in the common carotid artery [39]. These studies demonstrate that WSS measured from *in vivo* data vary throughout the vasculature. In addition, a study by Oshinski *et al.* [42] used MR-measured velocity profiles to determine a mean average WSS of 0.8±0.41 Pa in the common carotid artery of patients without disease. The mean WSS determined for the examined vertebrobasilar confluences ranges between 4.5 and 9.2 Pa. It is important to note that the WSS calculated in this study is calculated for the entire rigid model surface from the simulated flow field, not at a discrete location in a single artery from *in vivo* data acquired at the location.

Previously, oscillatory shear was shown to correlate with intimal thickening [6], [29], [43] and endothelial permeability [44]. In four of the five vertebrobasilar models, OSI foci were present at or near the confluence apex (Figure 8), which Ravensbergen *et al.* [3] identified as a site of atherosclerosis development. The OSI contours and WSS distributions did not have the same level of agreement as shown in He and Ku [29], although the OSI foci tended to correspond with low WSS regions. The small fraction of model surface with OSI ≥0.005 suggests that flow reversal occurs fleetingly or in focalized areas in these models. In branching vasculature, regions of adverse pressure gradient develop that can lead to reversed flow. These branch points are absent in the merging geometry of the vertebrobasilar system.

### Conclusions

In this paper, we describe the use of high-resolution MR angiography and *in vivo* quantitative flow measurements by PCMR in conjunction with CFD to develop subject-specific models of the VBS for non-invasively characterizing the physiological hemodynamics of the confluence. It is the first report of WSS distributions in the vertebrobasilar systems of healthy young adult subjects, and we have demonstrated that flow features (e.g. velocity profiles, WSS distributions) in these models are related to both the geometry and the relative flow rates of the system, which vary between normal subjects. The influence of geometrical features on the WSS distributions transcends inter-subject geometry differences. Further, the vertebrobasilar system is unique in the human body because it is the location where two large arteries merge into another vessel. Thus, understanding hemodynamics of this native, successful merge may offer insight for improved design of biomedical devices that involve merging flow (e.g. grafts, pumps).
